# Preventing and Managing Iatrogenic Dry Eye Disease during the Entire Surgical Pathway: A Study Focusing on Patients Undergoing Cataract Surgery

**DOI:** 10.3390/jcm13030748

**Published:** 2024-01-27

**Authors:** Giuseppe Giannaccare, Stefano Barabino, Antonio Di Zazzo, Edoardo Villani

**Affiliations:** 1 Eye Clinic, Department of Surgical Sciences, University of Cagliari, Via Università 40, 09124 Cagliari, Italy; 2 Ocular Surface and Dry Eye Center, ASST Fatebenefratelli-Sacco, Ospedale L. Sacco-Università di Milano, Via Giovanni Battista Grassi 74, 20157 Milan, Italy; stefano.barabino@unimi.it; 3 Ophthalmology, Foundation Campus Bio-Medico University Hospital, Via Alvaro del Portillo 200, 00128 Rome, Italy; a.dizazzo@unicampus.it; 4 Department of Clinical Science and Community Health, University of Milan, Eye Clinic San Giuseppe Hospital, IRCCS Multimedica, Via San Vittore 12, 20123 Milan, Italy; edoardo.villani@unimi.it

**Keywords:** dry eye disease (DED), meibomian gland dysfunction (MGD), ocular surface disease, surgery, cataract surgery, outcomes

## Abstract

Patient expectations for cataract surgery are continuously increasing, and dry eye disease (DED) represents a major cause of patient dissatisfaction in eye surgery. The present opinion paper aims to provide useful insights to improve the entire pathway of a patient undergoing cataract surgery, from the preoperative setting to the postoperative one. The available evidence from main clinical trials published on this topic is presented in association with experience-based points of view by the authors. Ocular surface disease (OSD) is common in patients presenting for cataract surgery, and more than half of these patients have DED and meibomian gland dysfunction (MGD), even in the absence of symptoms. Therefore, there is a need to encourage preoperative assessments for the risk of DED development or worsening in all patients as a routine approach to cataract surgery. New all-in-one diagnostic machines allow for fast and noninvasive screening of the ocular surface status. Once a preoperative diagnosis of DED/OSD is reached, ocular surface optimization should be obtained before surgery. In the case of unresolved OSD, the decision to delay surgery should be considered. The surgical procedure can be optimized by avoiding large incisions, limiting microscope light intensity and exposure, and avoiding an aspirating speculum or preserved eye drops. Postoperatively, the continued avoidance of preserved agents is advisable, as well as a limited exposure to epitheliotoxic antibiotics and nonsteroidal anti-inflammatory drugs. Short-term, preservative-free, soft corticosteroids may be useful for patients with extensive or persistent inflammation.

## 1. Introduction

Cataract surgery is among the most frequently performed procedures worldwide. Although it is cost-effective with a significant socioeconomic impact, increasing patients’ economic productivity, social autonomy, and quality of life, it is not a risk-free procedure, and undesired effects or iatrogenic conditions may occur, even after an uneventful surgery [1,2]. The most frequent postoperative complaints are related to ocular surface disturbances characterized by a wide range of symptoms, including a foreign body sensation, photophobia, burning, fluctuating vision, and epiphora [3]. This finding is not surprising, since most surgical procedures are performed on the older population that is characterized by a more frequent presence of ocular surface impairment. In fact, the prevalence of dry eye disease (DED) increases with age. In a cross-sectional survey conducted in the United States, the prevalence rate was found to be 2.7% in the age group of 18–34 years, while the prevalence rate was 18.6% in patients older than 75 years [4]. In another cross-sectional survey conducted in Canada, the prevalence rate was significantly higher (24.7%) in adults aged 55–64 years compared to that of adults aged 25–34 years (18.4%) [5]. Besides pre-existing DED, a variety of surgery-related parameters, such as the use of the traumatic lid speculum, the prolonged exposure to the operating microscope light, the corneal incisions, and the toxicity from perioperative topical therapies, among others, have roles in the occurrence of postoperative DED [6]. Since the signs and symptoms of ocular surface dysfunction are often poorly correlated in patients with DED, patient-reported symptoms or history cannot be used as the only tool to screen patients’ ocular surface status before surgery. Furthermore, a significant percentage of patients, especially older ones with severe vision impairment due to cataract, may not feel compelled to report ocular surface discomfort symptoms during routine preoperative evaluations. Therefore, easy, quick, and noninvasive diagnostic tools should be employed in addition to conventional subjective questionnaires to screen patients undergoing surgery to identify both subjects who are at risk and patients with alterations in their ocular surface status who require more in-depth evaluations and possibly preoperative management.

Besides cataract surgery, refractive, glaucoma, and eyelid surgeries can also determine newly developed DED or worsen pre-existing DED according to different mechanisms of action. For instance, most patients undergoing laser in situ keratomileusis (LASIK) complain of DED symptoms, especially in the early postoperative period. Post-LASIK DED usually peaks in the first few months after surgery, and then symptoms begin to improve in most patients 6–12 months postoperatively [7]. The term STODS (Surgical Temporary Ocular Discomfort Syndrome) has therefore been coined to describe the transitory perturbations of the ocular surface induced by surgery or laser surgery. However, in some cases, the insults result in a chronic ocular surface disease named SCODS (Surgical Chronic Ocular Discomfort Syndrome) [8].

Currently, patient expectations for cataract and refractive surgeries are higher than ever. Overall, the incidence of postoperative DED and the perception of dryness as a surgical complication could be reduced if information, detection, and guided optimization or treatment are provided preoperatively, keeping patients 20/Happy.

The aim of the present opinion paper is to provide useful insights for diagnosing and treating DED before, during, and after cataract surgery in order to optimize the entire pathway of the surgical patient.

## 2. Evaluating Patients before Surgery

In the PHACO study, even though upwards of 60% of routine patients screened for cataract surgery were asymptomatic, 50% of them presented central corneal fluorescein staining [9]; in another study, the prevalence of ocular surface disease in patients presenting for cataract surgery was higher than 80%, and more than 50% of asymptomatic patients had abnormal tear osmolarity or matrix metalloproteinase-9 level [10]. Furthermore, it has been reported that 52% of patients undergoing cataract surgery have clinical signs of meibomian gland dysfunction (MGD), the most common subtype of DED [11]. More recently, a Norwegian study employing the DEWS II criteria found a percentage of 55% of DED in patients scheduled for cataract surgery; the authors highlighted that DED was associated with the female sex, and no correlation was confirmed between signs and symptoms of DED [12].

Despite all the above-mentioned data, an educational gap still exists between the awareness of DED effects on surgical results and the efforts of ophthalmologists to cope with this issue. A recent annual ASCRS clinical survey reported that despite more than 90% of respondents feeling that DED affected patient satisfaction after cataract surgery, only 10% of them were using diagnostic testing in their routine preoperative assessments [13]. The most reasonable explanation is the common perception that preoperative ocular surface evaluation may be cumbersome, increasing the workup time. The recent rapid rise in commercially available noninvasive diagnostic tools for DED opens a new perspective in the screening of patients undergoing ocular surgery. These devices offer the advantage of obtaining automated results, avoiding subjective bias; moreover, since these examinations are noninvasive, they do not alter the results of subsequent examinations, representing useful tools for screening healthy subjects from patients affected by DED or those at risk for DED. Finally, using a comprehensive ocular surface workup may increase diagnostic accuracy to diagnose DED and monitor its course after therapies [14]. Integrating noninvasive ocular surface diagnostics in routine preoperative practice as a minimal workup for screening the eventual presence of pre-existing DED has little appreciable effect on patient turnover and doctor workload, while aiding in a rapid and reliable examination of the ocular surface status. In the case of pathological results from the screening or in patients with already-diagnosed DED, more in-depth examinations should be performed to identify the DED subtype and severity, such as corneal and conjunctival staining, Schirmer’s test, break-up time tests, meibum expression testing, corneal sensitivity, and point-of-care tests such as matrix metalloproteinase (MMP9) and tear osmolarity. Among noninvasive ocular parameters, the noninvasive break-up time, tear meniscus height, infrared meibography, and bulbar redness are the most studied ones. The use of optical coherence tomography allows for the determination of more detailed characteristics of the tear meniscus, such as the turbidity and area occupied by particles. Both values are higher in patients with MGD compared to controls [15].

## 3. Treating Patients before Surgery

Different approaches can be employed for the preoperative treatment of the ocular surface in patients undergoing surgery, such as (i) treating only patients with pre-existing ocular surface abnormalities; (ii) treating patients who are at risk for developing postoperative DED; and (iii) treating all patients regardless of their risk factors and impairment of the ocular surface system.

The least invasive and most used treatments in patients with pre-existing DED include over-the-counter tear substitutes and lubricating ointments. Prescription options for DED treatment include drugs, such as corticosteroids, cyclosporine, or lifitegrast. Thermal pulsation entails warming the eyelids at 40–42 °C with the goal of facilitating the expression of the meibum from the glands. A variety of devices using this technology are available on the market [16]. An improvement in DED and irritation symptoms, break-up time, and meibomian gland secretion has been reported after the implementation of these therapies. Finally, intense pulsed light leads to the coagulation and thrombosis of blood vessels, decreased abnormal blood vessel growth in the glands, greater oil secretion, and the destruction of inflammatory mediators. Reduced redness, vascularity, and increased meibum viscosity have been reported after intense pulsed light treatment [17]. Furthermore, a recent systematic review indicated that a proper treatment of DED before cataract surgery is also beneficial for the refractive outcomes of the procedure, significantly reducing postoperative errors [18]. In fact, diagnosing ocular surface disease in cataract surgery candidates is critical to optimize postoperative outcomes, because the tear film is an important component of the ocular diopter. In eyes with a healthy tear film, there may be a minimal power difference of 0.1 D between blinks. In people with an unstable tear film, there may be a variation of more than 1.0 D, which can correspond to an error of the same magnitude in lens power calculation and, thus, significantly impact vision.

To date, controversial results have been obtained by the few attempts of using prophylactically topical treatments for preventing DED postoperative occurrence. On one hand, the preoperative use of a short-term course of betamethasone 0.1% had no significant effect on postoperative DED indices [19]; on the other hand, two studies reported that the preoperative instillation of a tear substitute for periods of 1 week and 2 weeks reduced postoperative DED-related signs and symptoms to almost normal values [20,21]. Besides medical therapy, instrumental treatments have been used before cataract surgery with prophylactic purposes. A recent study demonstrated that prophylactic treatment with low-level light therapy (LLLT) in patients one week before and after cataract surgery can counteract the detrimental effects of surgery, not only avoiding the common postoperative decline in the ocular surface parameters, but also allowing for a significant improvement in ocular discomfort symptoms and tear stability compared to preoperative values [22]. Recently, Mencucci et al. and Zhao et al. conducted two studies on patients with MGD undergoing cataract surgery, evaluating the efficacy of vector thermal pulsation therapy, performed 5 weeks and one day preoperatively, respectively [23,24]. Both studies showed that this instrumental treatment was able to improve eyelid margin parameters and ocular discomfort symptoms. Another study from Park et al. included the same instrumental therapy for patients with either healthy ocular surfaces or MGD [25]. The most important finding was that patients without preoperative MGD benefited from receiving vector thermal pulsation therapy before surgery in terms of the meibomian quality, tear film stability, corneal staining, and DED symptoms.

Employing the newly developed ocular surface frailty index as a predictive tool for DED symptom onset after cataract surgery in the preoperative setting allows for the personalized assessment of patients at risk who could best benefit from a preventive treatment [26].

In rare cases, when a patient has severe unresolved ocular surface disease, it is advisable to postpone surgery until DED and/or MGD are satisfactorily managed [27]. In fact, persistent DED will contribute to vision instability, fluctuation, glare, and fatigue, which will drive both patient and physician dissatisfaction. Surgery in patients with untreated OSD may also be more prone to result in infection. For instance, patients with extensive corneal staining or damage should be carefully monitored and managed until they are more suitable for surgery; otherwise, severe complications could occur. When planning surgery in patients with DED symptoms, attention should be paid to environmental factors, since dryness, inflammation, and allergic reactions may all be exacerbated in late spring and summer. Surgery in patients with Sjögren’s syndrome or ocular graft versus-host disease should be considered as high-risk; these patients are prone to severe DED and tear-film instability. Treatment prior to surgery should typically include, in more severe cases, topical cyclosporine to improve tear production and decrease dry eye symptoms. Nerve damage, for example, from neurotrophic keratopathy or ulceration, also represents a decision point in the pathway to surgery. If surgery is undertaken in a sub-optimally managed eye, there is a risk of worsening damage to the trigeminal nerve, reducing corneal sensitivity and potentially resulting in corneal melting. Delaying surgery should also be considered in patients with active, unresolved MGD, primarily owing to the increased risk of bacterial infection during the surgery. Patients with MGD showed distinct clustering of the conjunctival sac bacterial community. Compared to the controls, at the phylum level, the presence of Firmicutes and Proteobacteria was significantly higher, while that of Actinobacteria was significantly lower in patients with MGD; at the genus level, the presence of Staphylococcus and Sphingomonas was significantly higher in patients with MGD, while that of Corynebacterium was significantly lower [28]. Prior to surgery, lid hygiene should be improved and maintained, and topical or systemic antibiotic treatment should be initiated when appropriate. A warm compress may also help to improve preoperative MGD in conjunction with established management strategies.

Regarding infection prophylaxis, antibiotics should be substituted with gentle antiseptics, which are less toxic to the corneal epithelium while avoiding the phenomenon of resistance [29]. Bacterial cleaning, to reduce the risk of infection, can be effectively performed with antiseptics and povidone-iodine cleansing.

## 4. Intraoperative Strategies

Contrasting results are available in the literature about the different impacts of femtosecond-laser-assisted cataract surgery (FLACS) and manual cataract surgery (MCS) on ocular surface parameters. Yu et al. reported that, even though both methods worsened DED postoperatively, FLACS had a higher risk for staining and DED symptoms, and patients with preexisting DED receiving FLACS had more severe ocular surface staining than those with MCS [30]. Another study by Shao and co-authors reported statistically significant differences only in the early stage, with FLACS having greater effects on CFS and DED symptoms [31]. The detrimental effect of laser may be related to the negative pressure of the suction ring and aspirating speculum, injuring the limbal stem cells, conjunctival epithelium, and goblet cells; altering mucin secretion; and increasing inflammation [32].

A recent meta-analysis comparing the two techniques reported that FLACS had higher severities on the ocular surface parameters, but most were not statistically significant. The impact was approximately the same three months postoperatively [33].

No clear evidence exists about the influence of various locations of corneal incisions on the development of DED, while a grooved incision seems to aggravate it during the early postoperative course [34]. On the contrary, studies agreed that phototoxicity from microscope light exposure, an increased duration of surgery, and a longer phacoemulsification time are contributory factors for DED after ophthalmic surgery [34,35]. 

An Intracameral mydriatic and anesthetic combination (Mydrane, Laboratoires Théa, Clermont-Ferrand, France) that can provide rapid and stable mydriasis and sustained intraocular anesthesia during cataract surgery shows a good safety profile and few toxic side effects compared to the standard protocol involving the use of eye drops, ensuring better optical quality and tear film stability [36].

During the surgery, the application of a polysaccharide blend of hydroxypropyl methylcellulose, xantham gum, and carrageenan (EyeDRO, Alchimia, Padua, Italy) on the corneal surface can provide a better coating compared to irrigation with a balanced salt solution, thus allowing ocular surface parameters and symptoms to return to preoperative values in a shorter period [37].

In terms of intraocular lens selection, caution is advised with multifocal lenses due to the potential inaccuracy of refractive power calculations in patients with more severe DED.

## 5. Postoperative Management

Generally, homeostasis indicators such as corneal sensitivity, tear film stability, and an average density of goblet cells are reduced at 1 day postoperatively, with a peak at 7 days, followed by a progressive recovery. Dry eye symptoms have been reported to persist for 1–3 months postoperatively, while in the worse scenario, the ocular surface does not recover until 6 months postoperatively. As a rule, increasing the frequency of postoperative follow-up visits can help determine patient progress and assess any need for adjustments to the treatment strategy. First, MGD should be considered and addressed, as it represents an important but frequently underestimated and undertreated factor that contributes to the vicious cycle of postoperative DED. Furthermore, it may be aggravated by cataract surgery. There are several clinical interventions for MGD, including eyelid hygiene, warm compresses, meibomian gland expression, omega-3 supplementation, and oral antibiotics [6]. A variety of topical treatments are available for postoperative DED, and with a better understanding of its pathophysiology, the optimal intervention has shifted from simply hydrating the ocular surface with artificial tears to applying eye drops with lubricating and cell-binding properties. Tear substitutes may play an important role in achieving the control of the inflammatory process in postoperative DED, improving tear fluid clearance and reducing the concentration of pro-inflammatory cytokines. Their use should be recommended at least during the first month after phacoemulsification in all patients, being able to improve tear stability, corneal staining, and DED symptoms postoperatively [38]. In a prospective, randomized, open-label, comparative study by Cagini and co-authors, an improvement was observed for clinical assessments and subjective ocular symptoms associated with DED at weeks 2 and 4 postoperatively for patients using a fixed combination of unpreserved trehalose and hyaluronic acid (HA) eye drops, which were statistically significant at week 4. There was also a statistically significant difference between the treatment groups at week 4 in favor of trehalose and HA compared to 0.9% sodium chloride for all measures, except for the Schirmer test [39]. In another prospective study by Mencucci and colleagues, the HA/trehalose ophthalmic solution effectively reduced post-cataract surgery DED signs and symptoms in patients with mild/moderate DED, particularly if also administered in both the preoperative and postoperative periods [40]. Eyelid scrubbing with eye shampoo containing tea tree oil could support DED treatment after cataract surgery, especially thanks to the decrease in the number of Demodex [41].

Postoperative care implies the use of topical antibiotics and nonsteroidal anti-inflammatory drugs (NSAIDs). The use of these drugs is associated with epithelial toxicity and worsening of goblet cell density. Therefore, in all cases of postoperative DED, epitheliotoxic antibiotics and NSAIDS should be avoided or used with caution and/or for a short duration. The use of corticosteroids has been demonstrated to ameliorate the signs and symptoms associated with DED as well as to prevent disease exacerbation. A review highlighted the differences existing among topical corticosteroids regarding the incidence of side effects and the evidence of efficacy. Good-quality evidence showing a minimal effect on intraocular pressure was reported for loteprednol etabonate and fluorometholone [42]. More recently, hydrocortisone was introduced in the market, and currently, its use is highly indicated for patients with DED, in which a long-lasting anti-inflammatory treatment is advisable [43]. Although this treatment can be considered safer than other types of corticosteroids, it is mandatory to check the intraocular pressure and the lens status during the treatment regardless of the molecule used. In a prospective study, patients with chronic DED and ocular surface inflammation received a preservative-free hydrocortisone, which resulted in reduced ocular inflammation and a decreased symptom score with no change in the intraocular pressure [44]. A retrospective review demonstrated that the topical application of the same preservative-free solution twice daily for 2 weeks significantly improved the clinical signs and symptoms in patients with mild to moderate DED [45]. The postoperative use of 3% diquafasol has been investigated after cataract surgery in two studies including, respectively, patients without or with pre-existing DED. In the former study, diquafasol allowed for a higher improvement in the break-up time and lipid layer thickness compared to 0.1% HA [46]; in the latter study, the tear break-up time, ocular discomfort symptoms, corneal staining score, lid margin abnormality, and meibum quality improved over time in patients using preservative-free diquafosol [47]. The addition of a systemic re-esterified triglyceride form of omega-3 fatty acids to tear substitutes in patients complaining of new-onset DED 1 month after uncomplicated cataract surgery allowed for the significant reduction in fluorescein corneal staining, subjective symptom scores, and matrix metalloproteinase-9 (MMP-9) level [48].

Although the presence of preservatives (in particular, benzalkonium chloride) in multidose formulations of topical ophthalmic medications is crucial for maintaining sterility, they can be toxic to the ocular surface. These adverse effects become more problematic with prolonged use or even with acute exposure in a clinically impaired ocular surface (DED, allergic reactions, and blepharitis); thus, preservative-free therapy should be prioritized in these cases to spare the ocular surface system [49]. In a recent open-label, prospective, randomized, comparative clinical trial, patients who were not previously affected by DED were assigned to receive either preservative-free or preserved dexamethasone 0.1% eye drops for 2 weeks after a standard phacoemulsification procedure. At week 2, a significant increase in corneal staining scores and foreign body sensation was observed only for the group receiving the preserved therapy [50]. In another randomized clinical trial, patients who were treated with preservative-free sodium hyaluronate 0.1% and preservative-free fluorometholone 0.1% eyedrops four times a day in the first month and twice a day in the second month had better values for the symptom score, break-up time, Schirmer test, fluorescein staining score, impression cytology, and goblet cell count compared to those receiving preserved formulations according to the same schedule [51].

## 6. Discussion

Although modern cataract surgery has been recognized as one of the most promising surgical procedures, postoperative outcomes can be negatively affected by DED onset or exacerbation [52]. Even though a variety of surgery-related factors have been associated with the development of iatrogenic DED, preoperative ocular surface impairment represents the most common causative factor that can be modifiable if promptly diagnosed and treated. Recognizing that patients may have subclinical ocular surface alterations is important because surgery is likely to expose DED symptoms that were unidentified in preoperative examination and exacerbate asymptomatic DED by increasing tear film instability. Surgical trauma can increase the production of free radicals, proteolytic enzymes, and inflammatory cytokines that can alter the ocular surface, and it is linked to neuropathy associated with the corneal incision during cataract surgery. For this task, a history of previous symptoms, lifestyle, work habits, contact lens intolerance, allergy, comorbidities, and medication use (diuretics, anticholinergics, antidepressants, antihistamines, or isotretinoin) should be investigated. In fact, some risk factors are modifiable and can be addressed, at least partially, before surgery. Validated questionnaires like the Ocular Surface Disease Index or Dry Eye Questionnaire are useful to preoperatively pick up symptomatology. An evaluation of the eyelids, including the meibomian glands, blink patterns, tear film, conjunctiva, and cornea, is mandatory. In particular, routine tests include the Schirmer test, tear break-up time, and ocular surface staining using fluorescein or lissamine green. New all-in-one diagnostic platforms allow for the quick and noninvasive calculation of the tear meniscus height, noninvasive break-up time, meibomian gland loss, and ocular redness. Few of them can perform tear film interferometry measuring the thickness and the break-up of the lipid layer. Point-of-care tests (e.g., MMP-9) can be used to quantify the inflammatory markers related to ocular surface inflammation. More advanced diagnostic modalities, such as in vivo confocal microscopy, can delineate cellular changes at the level of sub-basal nerve plexus like micro-neuromas, irregular branching, and an increased density of dendritic cells. If DED is already present preoperatively, it should be addressed and hopefully controlled before proceeding with surgery. Overall, the stepwise approach is not different from conventional DED, with the only exception being that a more aggressive approach is usually justified to improve the ocular surface status as soon as possible to avoid the delay of the surgery. Priority should be given to preservative-free eye drops both before and after surgery thanks to their ability to reduce dry eye symptoms. Increasing evidence shows a potential role of prophylactic treatment in the form of both medical and instrumental therapies in otherwise healthy patients scheduled for ocular surgery. Tear substitutes and, in selected cases, soft corticosteroids can support the ocular surfaces of patients during the entire pathway, from the preoperative setting to the postoperative one.

The main limitation of this paper is related to its narrative nature that lacks a systematic approach. However, our position paper provides advice and guidance on the main aspects regarding ocular surface health and disease during the entire surgical pathway of a patient.

In conclusion, the diagnosis and management of DED in the pre-, peri-, and postoperative phases of the surgical pathway is mandatory to contribute to a healthier ocular surface. Thorough preoperative optimization with timely surgery increases the likelihood of avoiding ocular surface complications, providing more stable vision and greater patient satisfaction.

## Data Availability

No new data were created or analyzed in this study. Data sharing is not applicable to this article.
